# Recent Advances in Function-based Metagenomic Screening

**DOI:** 10.1016/j.gpb.2018.01.002

**Published:** 2018-12-29

**Authors:** Tanyaradzwa Rodgers Ngara, Houjin Zhang

**Affiliations:** Department of Biotechnology, College of Life Science and Technology, Huazhong University of Science and Technology, MOE Key Laboratory of Molecular Biophysics, Wuhan 430074, China

**Keywords:** Metagenomics, Function-based screening, Agar plate screening, Microfluidics, FACS-based screening

## Abstract

Metagenomes from uncultured microorganisms are rich resources for novel enzyme genes. The methods used to screen the metagenomic libraries fall into two categories, which are based on sequence or function of the enzymes. The sequence-based approaches rely on the known sequences of the target gene families. In contrast, the function-based approaches do not involve the incorporation of metagenomic sequencing data and, therefore, may lead to the discovery of novel gene sequences with desired functions. In this review, we discuss the function-based screening strategies that have been used in the identification of enzymes from metagenomes. Because of its simplicity, agar plate screening is most commonly used in the identification of novel enzymes with diverse functions. Other screening methods with higher sensitivity are also employed, such as microtiter plate screening. Furthermore, several ultra-high-throughput methods were developed to deal with large metagenomic libraries. Among these are the FACS-based screening, droplet-based screening, and the *in vivo* reporter-based screening methods. The application of these novel screening strategies has increased the chance for the discovery of novel enzyme genes.

## Introduction

The total number of microbial cells on Earth is estimated to be around 10^30^ [1]. The largest proportion of the microbial population is made up of prokaryotes that comprise 10^6^–10^8^ separate species [2]. It is widely accepted that prokaryotes possess unique microbial diversity and represent a largely-unexplored biological and genetic reservoir that can be exploited for novel enzymes with unique metabolic capabilities [3], [4]. However, conventional techniques used in the laboratory to culture bacteria are often inefficient and limited as most of bacteria taken from environmental samples are currently unculturable [4], [5]. This could be attributed to a number of different factors such as alterations in atmospheric oxygen levels, osmotic conditions, specific nutrients required for survival, as well as pH and temperature conditions [6]. Hence the routine culture environments provided in the laboratory impose a selective pressure that would prevent the majority of microbes from growing [5], [6]. Therefore, a suite of culture-independent techniques is essential to understand the population structure, ecological roles, evolution, and genetic diversity of unculturable microbes found in natural environments [5], [6], [7].

**Figure 1:**
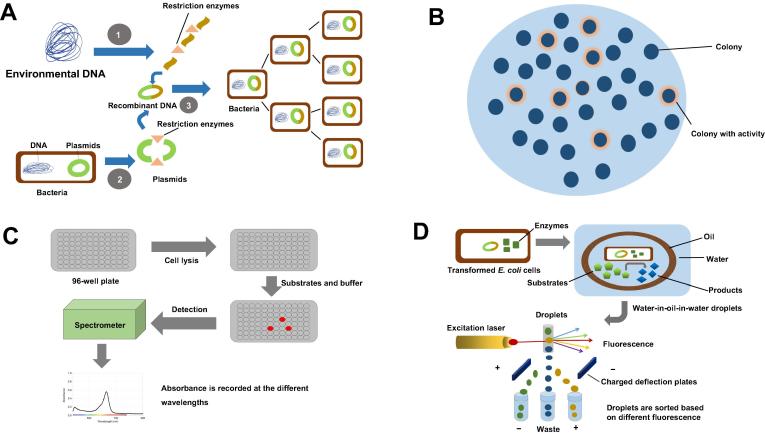
**An overview of metagenomic screening approaches** **A.** Steps involved in the construction of a library from environmental metagenome. **B.** Agar plate activity screening. **C.** Microtiter plate screening. **D.** Microfluidics coupled with FACS. FACS, fluorescence-activated cell sorting.

Metagenomics holds great promise for tapping the rich genetic resources in the uncultured microorganisms, by skipping culturing microorganisms and isolating genomic DNA directly from an environmental sample [8]. The rich genetic resource in the resulting metagenomic libraries can be explored in two ways: sequence-driven and function-driven screening [3], [9]. The sequence-based screening is used more frequently than the function-based approaches, due to the easy access to the metagenomics sequencing data and many software available for data analysis. However, sequence-based approaches have inherent drawbacks, with their effectiveness largely affected by the accuracy of genome annotation and the completeness of the data available [10]. These approaches reply on the algorithms available and information present in databases to infer the functions of newly-discovered genes. Thus they may not work well if the sequence similarity does not correspond to a functional relationship, or if the novel gene has only a weak similarity to any genes whose products have been examined biochemically, or if a particular gene is able to carry out numerous functions in the cell [10], [11]. Therefore, function-driven screening is the preferred approach when it comes to discovering genes with novel functions or exploring the sequence diversity of protein families with certain functions [5]. The basic procedure of the functional metagenomic screening, which includes the construction of metagenomic libraries and the different assay techniques employed to identify novel genes with desired functions, is illustrated in Figure 1.

This review is directed toward a concise synopsis of the different function-driven metagenomics screening techniques that have recently been used. A variety of novel genes encoding novel enzymes have been discovered in this way. Nonetheless, some inherent limitations of conventional function-driven screening strategies call for attention, such as high time consumption, low hit rate, and labor-intensive operations [3], [8]. To cope with these problems, several high-throughput screening strategies have been developed during the past two decades, such as microfluidics-based screening and reporter-based screening [10], [12]. The metagenomic studies and corresponding functional screening strategies discussed in this review are summarized in Table 1.

**Table 1:** **Recent examples of functional screening strategies employed to obtain metagenome-derived biocatalysts**

**Screening approach**	**Target gene**	**Detection method**	**Inducer**	**Source**	**Host, vector**	**Ref.**
Agar plate screening	Proteases	Phenotypical detection	1% skim-milk	Goat skin	*E. coli*, plasmid	[13]
Agar plate screening	Proteases	Phenotypical detection	1% skim-milk	Desert sands	*E. coli*, plasmid	[14]
Agar plate screening	Proteases	Phenotypical detection	AZCL-casein	Deciduous forest soil	*E. coli*, plasmid	[15]
Agar plate screening	Esterases	Phenotypical detection	1% Tributyrin	Marine mud	*E. coli*, plasmid	[16]
Agar plate screening	Esterases	Phenotypical detection	X-caprylate	Vegetable soil	*E. coli*, plasmid	[17]
Agar plate screening	Esterases	Phenotypical detection	α-Naphtyl-acetate, fast blue RR	Salted shrimp	*E. coli*, plasmid	[18]
Agar plate screening	Esterases	Phenotypical detection	1% Tributyrin	Alluvial soil	*E. coli*, plasmid	[19]
Agar plate screening	Esterases	Phenotypical detection	Dimethyl phthalate	Biofilm waste treatment plant	*E. coli*, fosmid	[20]
Agar plate screening	Lipase	Phenotypical detection	1% Tributyrin	*Cymbastela concentrica*, *etc.*	*E. coli*, fosmid	[21]
Agar plate screening	Lipase	Phenotypical detection	1% Tributyrin	Marine sponge (*Haliclona simulans*)	*E. coli*, fosmid	[22]
Agar plate screening	Lipase	Phenotypical detection	1% Tributyrin	Marine sponge (*Ircinia* sp.)	*E. coli*, plasmid	[23]
Agar plate screening	Lipase	Phenotypical detection	1% Tributyrin	Biomass	*E. coli*, cosmid	[24]
Agar plate screening	Cellulase	Phenotypical detection	Carboxymethyl cellulose	Coral (*Siderastrea stellata*)	*E. coli*, fosmid	[25]
Agar plate screening	Cellulase	Phenotypical detection	AZCL-HE-cellulase	Brown alga (*Ascophyllum nodosum*)	Yeast, plasmid	[26]
Agar plate screening	Starch hydrolyzing enzyme	Phenotypical detection	Starch, Lugol solution	Acid mine drainage	*E. coli*, plasmid	[27]
Agar plate screening	β-Glycosidases	Phenotypical detection	X-gal	Baltic Sea water	*E. coli*, plasmid	[28]
Agar plate screening	β-Glycosidases	Phenotypical detection	4-Nitorphenyl-β-d-glucoside	Alkaline polluted soil (Guangxi)	*E. coli*, plasmid	[29]
Agar plate screening	β-Glycosidases	Phenotypical detection	AZCL-xylan, xyloglucan	Cow dung	*E. coli*, phage	[30]
Agar plate screening	Pectinases	Phenotypical detection	Pectin	Forest soil (Western Ghats, India)	*E. coli*, plasmid	[31]
Agar plate screening	Tannase	Phenotypical detection	X-caprylate	Cotton field soil	*E. coli*, plasmid	[32]
Agar plate screening	Nuclease	Heterologous complementation	Hydrogen peroxide	Soil (Joao, Brazil)	*E. coli*, plasmid	[33]
Agar plate screening	RNase H	Heterologous complementation		Leaf and branch compost	*E. coli*, plasmid	[34]
Agar plate screening	Dioxygenases	Phenotypical detection	Indole	Aromatic compounds polluted soil	*P. putida*, phage	[35]
Agar plate screening	Dioxygenases	Phenotypical detection	Catechol	Activated sludge	*E. coli*, plasmid	[36]
Agar plate screening	Di-chlorophenol hydroxylase	Phenotypical detection	3,5-Dichlorocatehol	Polychlorinated biphenyl contaminated soil	*E. coli*, plasmid	[37]
Agar plate screening	Polyhydroxyalkanoate synthase	Heterologous complementation		Sandy loam surface soil	*S. meliloti*, cosmid	[38]
Agar plate screening	Laccase	Phenotypical detection	2,6-DMP, *etc.*	Mangrove soil	*E. coli*, plasmid	[39]
Agar plate screening	DNA polymerase	Heterologous complementation	Cold sensitive mutant strain	Glacial ice	*E. coli*, fosmid	[40]
Agar plate screening	Hydrogenase	Heterologous complementation	Freshwater enrichment	Not stated	*E. coli*, plasmid	[41]
Agar plate screening	Genes resistant to toxic elements	Phenotypical detection	pH 1.8	Acidic water (Tinto river)	*E. coli*, plasmid	[42]
Agar plate screening	Genes resistant to toxic elements	Phenotypical detection	Different number of antibiotics	Human fecal	*E. coli*, fosmid	[43]
Agar plate screening	Genes resistant to toxic elements	Phenotypical detection	Different number of antibiotics	Soil	*E. coli*, plasmid	[44]
Agar plate screening	Genes resistant to toxic elements	Phenotypical detection	Several antibiotics	Dairy cow manure	*E. coli*, fosmid	[45]
Agar plate screening	Genes resistant to toxic elements	Phenotypical detection	Several antibiotics	Cheese food matrix	*E. coli*, fosmid	[46]
Agar plate screening	Genes resistant to toxic elements	Phenotypical detection	Chloride salts	Surface water (Mississippi River)	*E. coli*, fosmid	[47]
Agar plate screening	Genes resistant to toxic elements	Heterologous complementation	Acrylate	Wastewater treatment plant	*E. coli*, cosmid	[48]
Microtiter plate screening	Cellulase	Absorbance measurement	Dinitrophenol-cellobioside	Soil, Buffalo rumen, *etc.*	*E. coli*, fosmid	[49]
Microtiter plate screening	Esterase	Absorbance measurement	Nitrophenyl acetate	Oil reservoir	Not stated, fosmid	[50]
Microtiter plate screening	Plant polymer decomposing enzymes	Absorbance measurement	Multiple substrates	Leaf compost	*E. coli*, fosmid	[51]
Microtiter plate screening	Laccase, exochitinase, *etc.*	Absorbance measurement	ABTS, methylumbelliferone	Forest soil (Morvan, France)	Not stated	[52]
SIGEX-FACS	Aromatic hydrocarbon catabolic operons	Fluorescence	Benzoate, naphthalene	Ground water contaminated with crude oil	*E. coli*, plasmid	[53]
SIGEX-FACS	Transcriptional regulators	Fluorescence	Salicylate, *etc.*	Ground water contaminated with crude oil	*E. coli*, plasmid	[54]

SIGEX-FACS	Aromatic hydrocarbon induced genes	Fluorescence	Salicylate Benzoate, *etc.*	Soil contaminated with polyaromatic hydrocarbon	*E. coli*, plasmid	[55]

GESS-FACS	Phenol generating enzymes	Fluorescence	p-Nitrophenyl	Sea tidal flat sediments	*E. coli*, plasmid	[56]
GESS-FACS	Lipase, cellulase, *etc.*	Fluorescence	p-Nitrophenyl acetate, *etc.*	Ocean tidal flat sediment	*E. coli*, fosmid	[57]
GESS-FACS	Phosphatase	Fluorescence	p-Nitrophenyl phosphate	Ocean tidal flat sediments	*E. coli*, fosmid	[58]
GMD	Lipolytic enzymes	Fluorescence	Fluorescein dicaprylate	Soil (*Quercus serrata* forest)	*E. coli*, plasmid	[59]

GMD, FACS	Screening for antibiotics	Fluorescence	*S. aureus*	3 strains of *Staphylococcus* obtained from an ARS culture collection	*E. coli* *S. cerevisiae*, plasmid	[60]

Microfluidics (water in oil droplets), FACS	Hydrolases	Fluorescence	Sulfate monoester Phosphate triester	Variety of sources (soil, degraded plant material, cow rumen)	*E. coli*, plasmid	[61]

*Note*: AZCL, azurine-cross-linked; AZCL-HE, azurine-cross-linked hydroxyl-ethylcellulose; DMP, dimethoxyphenol; ABTS, 2,2′-azino-bis(3-ethylbenzothiazoline-6-sulfonate); GESS, genetic enzyme screening system; GMD, gel micro-droplet; FACS, fluorescence-activated cell sorting; SIGEX, substrate induced gene expression; ARS, agriculture research service.

## Agar plate screening

### Agar plate screening for hydrolytic enzymes

Functional screening of metagenomic libraries using agar plates provides a simple and straightforward approach to identify novel enzymes that function under diverse conditions. Numerous novel hydrolytic enzymes, including lipases, esterases, cellulases, proteases, laccases, glycosylases, nitrilases, and dehalogenases, have been identified using this method [62]. Assays used to discover these enzymes are based on the production of a chromophore or fluorophore in colonies incubated with a chromogenic or fluorogenic substrate. The throughput of agar plate based assays is usually 10^5^–10^6^ clones per day [63]. Despite the low throughput, agar plate screening has led to the successful isolation of a large number of unique enzymes from various environments. In the following section, we describe the approaches used to identify novel hydrolytic enzymes.

#### Proteases

The catabolism of proteins is catalyzed by proteases via cleaving the peptide bonds that link amino acids within proteins [64], [65], [66]. Screening of protease activity involves incubating 1% skim-milk on LB agar plates and a positive signal for protease activity is shown as halos due to the degradation of milk proteins [13], [14], [65]. A lot of proteases with desirable properties have been identified using this simple technique [64], [65], [66], [67]. For instance, a metagenomic library containing DNA from bacteria dwelling on goat skin was functionally screened for protease activity, which leads to the discovery of a serine protease enzyme with a high alkalinity tolerance [13]. A metagenomic library containing clones derived from a forest soil sample was functionally screened for protease activity on yeast extract and tryptone agar plates supplemented with azurine-cross-linked casein. The screening result in the discovery of a novel alkaline serine protease with high oxidant stability [15]. Similarly, activity screening of metagenome libraries of soil samples from Death Valley and Gobi deserts revealed two serine proteases with distinctly thermophilic profiles [14].

#### Esterases and lipases

Ester bonds can be synthesized and hydrolyzed by enzymes in carboxylic ester hydrolase class, which contains lipases and esterases [16], [68], [69]. Agar plate screening has been used to identify novel esterases [15], [19], [65], [69], [70], [71], [72], [73], [74], [75], [76], [77], [78] and lipases [67], [79], [80]. Detection of lipase or esterase activity is usually performed using tributyrin since hydrolysis of emulsified tributyrin leads to the formation of halos around positive colonies. To specifically detect lipase activity, LB agar media containing olive oil is used [68], [81], [82], with the addition of a fluorescent dye Rhodamine B. The orange fluorescence, from a complex of fatty acids and Rhodamine B, serves as a good indicator of lipase activity [62], [68].

A novel pyrethroid-hydrolyzing esterase enzyme, which has potential applications in insecticide production, was identified from a metagenomic library of a soil sample at vegetable garden [17]. A similar screening approach resulted in the identification of a highly organic solvent-tolerant and thermostable esterase [16]. When using fast blue RR staining to identify clones that exhibited esterase activity from a salted shrimp derived metagenomic library, a unique salt-tolerant esterase was identified [18]. Agar plate activity screening of an alluvial soil metagenomic library revealed the identity of two unique esterases that exhibited chloramphenicol reactivating activity [83], whereas two esterase with distinctly cold adapted activity were identified when activity-based screening was carried out with an arctic soil derived metagenomic library under low temperature [84]. Additionally, a substrate set, composed of di-butyl phthalate, di-propyl phthalate, and di-pentyl phthalate, was utilized to carry out activity-based screening of a wastewater-derived metagenomic library [20]. As a result, three novel phthalate esters hydrolase enzymes were discovered, which exhibited an adaptation to cold activity.

To identify lipases, metagenomic libraries derived from microbe societies associated with green alga (*Ulva australis*) and with a temperate marine sponge (*Cymbastela concentrica*) were functionally screened. This leads to the identification of three unique lipases that exhibited potential antibacterial properties [21]. Additionally, functional screening of a marine sponge (*Haliclona simulans*) derived metagenomic library revealed a lipase that adapted to high salinity concentrations [22]. Similarly, a lipase displaying alkalophilic activity was identified from the metagenomic library constructed from marine sponge *Ircinia* sp [23]. Following functional screening of metagenomic libraries derived from a cell culture enriched at hot and high pH condition, a thermo-stable alkaline lipase was discovered [24].

#### Cellulases

Cellulases are a group of enzymes which hydrolyze cellulose. Agar plate activity screening for cellulases and glycosyl hydrolases often involves the use of carboxymethyl cellulose as a substrate and Congo red dye as an indicator [85], with unstained haloes representing positive clones. Such method has been used to discover many novel cellulases from different environments, some of which are from unexpected locations with no selective pressure for cellulases [65], [67], [86], [87], [88], [89]. For instance, cellulases are found in a metagenomic library from coral *Siderastrea stellata* [25]. The coral is not a place you would expect to find cellulases, because it contains very little cellulosic substance. Other cellulases are found to have properties matching their surrounding environments. For example, a halo-tolerant and cold-active cellulase was identified in a metagenomic library constructed with microbial DNA from a cold-active marine brown alga *Ascophyllum nodosum* [26].

#### Xylanases

Xylanases are hydrolytic enzymes that degrade the linear polysaccharide beta-1,4 xylan into xylose, thus breaking down hemicellulose. Detection of xylanase activity is done using birchwood xylan as substrate and Congo Red as indicator dye, with positive clones identified by unstained halos. Identification of novel xylanases from metagenomics has been reported in a number of studies [90], [91], [92], [93], [94].

#### Starch hydrolyzing enzymes

Screening for starch hydrolyzing activity in the metagenomic library uses starch as substrate and Lugol solution (iodine) as the indicator dye, with positive clones identified as clear halo zones around the colonies [27], [95]. Agar plate activity screening for novel starch hydrolyzing enzymes, such as amylases, pullulanases, and glucoamylases, has been reported in many studies [65], [67], [95], [96]. These starch hydrolyzing enzymes show special characteristics adapted to the surrounding environmental elements, such as coldness and high pH. Some amylases with no sequence homolog to known proteins were found, which expands the sequence diversity of the amylase enzyme family. For example, function-based screening of an acid mine drainage-derived metagenomic library led to the identification of two amylases containing no known amylolytic domain [27]. It is noteworthy that these amylases do not share any sequence similarity with known amylases or glycoside hydrolase either. Therefore, they represent a new subgroup of the amylase enzyme family.

#### β-Glycosidases

β-Glycosidases are enzymes that cleave beta-glycosidic bonds [97]. Agar plate activity screening for β-glucosidase activity is carried out using ammonium ferric citrate-esculin, while p-nitrophenyl-β-d-xyloside (pNPX) is used to identify β-xylosidase [97], [98]. Using agar plate activity screening, novel β-glycosidase enzymes had been reported in several studies [29], [97], [98], [99], [100], [101]. A novel cold-active glycosidase that exhibited beta-glucosidase, beta-fucosidase, and beta-galactosidase activities was detected from of a metagenomic library derived from a marine sample [28]. Another novel β-glucosidase with both glycosidase and lipolytic activity was identified from clones of metagenomic library derived from an alkaline polluted soil sample [102]. Similar screening was also used to explore the microbiota in animal stomach samples. A screening of genes encoding fibrolytic enzymes resulted in the discovery of four protozoan glycoside hydrolases from a bovine ruminal protozoan-enriched metagenomic library [30].

#### Pectinases

Screening for pectinolytic activity is based on growing clones on pectin-containing LB plates using Ruthenium red solution as indicator, with positive clones shown as clear zones. Activity-driven analysis of clones from a metagenomic library obtained from a tropical forest soil sample led to the identification of a pectinase active at broad pH and temperature [31], indicating that metagenomes could be a useful source for pectinase discovery.

#### Tannases

Tannases catalyze the hydrolysis of digallate, which is found in many bacteria, such as those dwelling in rumen. Functional screening of tannases involves the use of isopropyl-β-d-thiogalactopyranoside (IPTG) as the inducer and X-caprylate as the indicator dye, with positive clones showing a distinctive blue color. Function-driven analysis of clones from a metagenomic library constructed with a cotton field soil sample resulted in the detection of a gene encoding tannase [32]. This tannase shows excellent resistance to salt concentration as high as 4 M NaCl. It also has a broad spectrum of substrates, making it a potential biocatalyst with potential industrial application.

#### Nucleases

Functional screening was also used to identify novel enzymes responsible for DNA repair that are vital in preserving the integrity of DNA [33]. Clones from the metagenomic library were analyzed through complementation assay using *Escherichia coli* strain DH10B, which is sensitive to hydrogen peroxide due to the lack of DNA repair gene encoding recA. A positive clone was identified based on the increased resistance to hydrogen peroxide treatment. The resulting gene encodes a novel salt-tolerant exo-nuclease with no significant similarity to any known nuclease.

Similarly, complementary genetic screening was employed to identify unique RNase H genes from a metagenomic library constructed from a leaf and branch compost [34]. Twelve unique genes encoding type 1 RNases H were identified, of which eleven genes showed 40%–72% protein sequence identities to those found in the National Centre for Biotechnology Information (NCBI) database. Interestingly, another enzyme identified in this study lacks a conserved DEDD/E active site motif, but still exhibited RNases activity, indicating distinctive catalytic mechanism.

### Agar plate activity screening for non-hydrolytic enzymes

Typically, the targets for metagenomics screening are various hydrolytic enzymes, due to the easy detection of the phenotypes, such as the formation of halos around the colonies. Occasionally, metagenomics screenings are used to screen non-hydrolytic enzymes as well.

#### Dioxygenases

Aimed to identify enzymes that are able to degrade aromatic compounds, Nagayama et al. performed an indigo-forming activity analysis on clones from a metagenomic library derived from an aromaticallypolluted soil sample. In this study, a gene encoding a multicomponent hydroxylase was found to be responsible for the phenol degradation [35]. A similar analysis was also performed to identify the novel enzymes able to carry out microbial degradation of aromatic compounds from sludge. Consequently, 38 new genes coding for extradiol dioxygenases were identified, forming a new subfamily of extradiol dioxygenases [36].

#### Dichlorophenol hydroxylases

Lu and colleagues collected an environmental sample from an area contaminated by polychlorinated bi-phenyl compounds for more than two decades and used it for the construction of a metagenomic library [37]. They used 3,5-dichlorocatechol to screen for chlorophenol hydroxylase genes and identified a 2.4-dichlorophenol hydroxylase with a *K*_m_ of 5 µM for 2,4-dichlorophenol and 6 µM for NADPH. This was the first report of identifying a unique *TfdB* gene using the functional metagenomic approach.

#### Polyhydroxyalkanoate synthases

The phenotypic complementation was employed when screening for novel genes encoding polyhydroxyalkanoate synthases in a metagenomic library from soil. Several genes with low identity to known genes that are involved in polyhydroxyalkanoate synthesis were isolated, which expands the diversity of this group of genes [38].

#### Laccases

Based on a metagenomic library constructed from a mangrove soil sample, function-driven screening resulted in the identification of a multi-copper oxidase with laccase activity [39]. This laccase shows good alkaline activity and good solubility, which set it apart from other laccases.

#### DNA polymerases

Complementation assay using *E. coli polA* mutant was employed in the screening of glacial ice derived metagenomic libraries. This mutant strain cannot survive at the temperature below 20 °C due to a mutation in the DNA polymerase gene. Therefore, during the screening process, only the cells carrying the metagenomic clones conferring DNA polymerase activity can survive at 18 °C. Such screening resulted in the detection of nine genes encoding DNA polymerases [40].

#### Hydrogenases

In an activity-based approach to identify an acid-tolerant hydrogenase from metagenomic libraries, the clones were transferred via tri-parental mating into a hydrogenase-deleted mutant *Shewanella oneidensis* and grown on freshwater medium [41]. The clones that exhibited hydrogenase activity were distinguished by the change in color of the media from yellow to colorless.

### Screening for the genes responsible for the resistance to toxic elements

The agar-plate activity screening method is often used to screen for the genes responsible for the resistance to the toxic elements such as antibiotic, extreme salt concentration, extreme pH, and heavy metals. Function-driven analysis of metagenomic libraries derived from rhizosphere and planktonic microbial communities revealed 15 novel genes with the encoded proteins conferring acid resistance [42]. Most products of these genes are putative or unknown proteins, which implies the unknown mechanism for acid resistance. Guazzaroni et al. also identified nine genes that were expressed at high levels in *P. putida* and *Bacillus subtilis*, which enhanced host cells’ ability to withstand extreme acidic conditions [42].

In a search for antibiotic resistance genes, seven different antibiotics were used to screen a fosmid library constructed from gut microbiota of four healthy humans, which resulted in the discovery of eight new antibiotic resistance genes [43]. Similarly, functional screening of clones from a soil metagenomic library revealed 41 novel genes that encode antibiotic resistance proteins across eight protein families [44]. In another study, metagenomic libraries derived from a cow manure sample were functionally screened for resistance to a number of antibiotics, resulting in the detection of 80 unique antibiotic enzymes together with a novel clade of chloramphenicol acetyl-transferases [45]. Similar screening was also performed with metagenomic libraries derived from food fermenting microbiota in the presence of a wide range of antibiotics. Novel kanamycin and ampicillin resistant clones originating from *Lactobacillus helveticus* and *Streptococcus salivarius* were reported [46]. Like the antibiotic resistant genes’ (ARGs) screening, metal resistance activity screening on metagenomic libraries derived from the Upper Mississippi River showed the highest frequency of clones with resistance to Mn^2+^ but no clone with resistance to Cu^2+^ [47]. In the quest to find unique genes that confer bacterial resistance to acrylate, activity screening of clones from a metagenomic library derived from a sewage treatment plant revealed three enzyme classes that conferred an acrylate-resistant (Acr^R^) phenotype [48].

## Microtiter plate screening

Agar plate screening approaches are simple to perform and provide an easy way to identify active clones. However, they have major setbacks, with low dynamic range and weak visualization of differences in catalytic rates of enzyme variants, and, therefore, difficulty in quantification [9], [12]. In order to improve sensitivity, a number of different approaches are applied. The most commonly applied screening strategy is based on microtiter plates, which involves incubation of bacterial culture with enzyme substrate in the micro-wells [9], [103]. Usually, a single colony or diluted cell culture containing several colonies is distributed into each individual well manually or through a liquid handling station. The cells are grown in microtiter plates and assessed in a second plate after cell lysis, with the original plates stored as back-up [9], [104]. With the occurrence of substrate conversion, a visual signal emerges, such as color or fluorescence, which is used to identify colonies expressing an enzyme with desirable properties. The throughput of microtiter plates has been enhanced through the incorporation of robotic technologies [104]. Also, fluorescence-activated cell sorting (FACS) has been used to distribute single cells into microtiter plates [105]. However, these kind of experiments require a strong prior knowledge on the best convenient substrates and also the chemistry on how it works. This presents a bottleneck and consequently, microtiter plate screening is not applicable in many occasions [104].

In a study, chromogenic dinitrophenol (DNP)-cellobioside was used as the substrate to identify cellulase activity [49]. The 384-well microplates allow for quantitative analysis since the plate reader can be used to measure the absorbance. Moreover, fosmid library was constructed to improve the efficiency and reliability of the activity-based screening. It was noticed that adding the inducer L-arabinose in the culturing media resulted in an increase in the copy number of the fosmid library by up to 100 folds in the *E. coli* cells while a single copy of the fosmid library was maintained during the growth period for stability [9].

In a metagenomic analysis of an oil reservoir metagenome sample, *E. coli* cells containing fosmid library were grown in microtiter plates. The cells in the duplicate plates were lysed, and the resulting supernatants were transferred to 384-well microtiter plates. The enzyme assays for short chain and long chain esterase activity were performed by adding the crude cell extracts to microtiter plates containing nitrophenyl acetate and nitrophenyl palmitate as substrate, respectively. One enzyme showing the highest activity on short chain ester substrate was identified as a novel esterase exhibiting high thermo-stability and high tolerance to metal ions and solvents [50].

A high-throughput screening technique was also developed for the identification of enzymes involved in starch, hemicellulose, cellulose, chitin, lignin decomposition, protein hydrolysis, and phosphate mineralization, using the multiple substrate approach to allow for concurrent identification of diverse activities essential during the various stages of biomass depolymerization [51]. Enzyme assays were performed on microtiter plates and resulted in the identification of phosphatases, carbohydrate hydrolase, and protease activity. It is noticeable that such a method requires the extensive use of sophisticated equipment, such as liquid handling system and automatic colony picker. This may limit its use in the labs without such setups.

To investigate the functions of microbial communities at different soil layers, special microtiter plates, Biolog Ecoplates, were used to study the metabolic profiles of the soil microbiome [52]. Uroz et al. analyzed metagenomic libraries derived from two acidic forest soil samples that were incubated with different carbon sources on Ecoplate microplates. They detected positive results by measuring color development at 590 nm on a microplate reader. In addition, functional screening for laccase, β-glucosidase, xylosidase, cellobiohydrolase and exochitinase activity were also performed by inoculating with relevant substrates on regular microtiter plates. It was found that the substrates that were readily metabolized differed between the two soil samples, suggesting that the microbial communities had a specialization in response to nutritional conditions [52].

## FACS-based screening

There are a number of challenges associated with the conventional function-driven screening approaches in the analysis of metagenomic libraries, such as low throughput, low hit frequency for positive clones, and increased evidence of catalytic promiscuity [3]. Moreover, these approaches are often labor intensive and time-consuming [7]. One solution to such problems is to use FACS to screen the libraries. FACS enables the selection of cells based on cell size, shape, and fluorescence [12], [59], [106], [107], [108]. FACS have many advantages: (1) it deposits single events into a variety of vessels quickly and accurately; (2) the laminar flow fluidics of FACS prevents disruption of cells during sorting; and (3) the contamination is limited because of the small volume of each droplet [105], [109], [110]. Due to its powerful sorting abilities, FACS is easily coupled to a number of different high-throughput screening methods such as droplet sorting and reporter-based screening. The reporter-based screening method depends on the expression of the reporter genes, such as GFP genes [12], [111]. In these systems, the metagenomic library is transformed into the host cells which harbor the reporter genes. Then the gene products of the metagenomic library activate the expression of reporter genes through transcriptional regulation or post-translational modifications [12].

A good example of reporter-based screening system is the substrate induced gene expression (SIGEX) system which is coupled with FACS to sort GFP-expressing *E. coli* cells that respond to the presence of aromatic compounds [53]. Using SIGEX, Uchiyama et al. identified numerous transcriptional regulators which turn on the report gene expression in response to aromatic compounds, such as salicylate, 3-methyl catechol, 4-chlorocatechol, and chlorohydroquinone [54]. In another study, several aromatic hydrocarbon (AH)-degrading genes were discovered using SIGEX in a metagenomic library derived from AH-contaminated soil sample [53]. Subsequently, the library was sequenced for contig assembly. The AH-degrading genes were mapped to the contigs, resulting in the identification of several complete operons involved in the AH metabolism [55]. This approach combines the SIGEX technique and the next-generation sequencing (NGS) technology, which is very instrumental in studying the operons or clusters in the unculturable microorganisms.

Similarly, a genetic circuit termed genetic enzyme screening system (GESS) was developed for the high-throughput function-driven analysis of unique phenol-generating enzymes from metagenomic libraries [56]. Through a phenol-binding transcriptional activator, phenol generated by these enzymes can trigger the expression of GFP reporter gene. Three such enzymes were identified, including alkaline phosphatase, lipase, and cellulose, with the help of phenolic substrates (phenyl phosphate, p-nitrophenyl acetate, and p-nitrophenyl-β-d-cellobioside) respectively [57]. Also, p-nitrophenyl phosphate (pNPP)-GESS was used to identify a novel psychrophilic alkaline phosphatase, together with the phenol-recognizing dimethylphenol regulator (DmpR) as the transcriptional activator [58].

## Microfluidics-based screening

In addition to the conventional FACS-based screening, a more advanced high-throughput screening strategy was developed which use microfluidic chips to generate monodispersed microdroplets [12], [112]. Microdroplets are produced in large numbers at speeds of thousands of droplets per second and a single droplet functions as a reaction chamber. Cells, enzyme variants, substrates and products are confined in the picoliter volume of the droplets, where reactions take place [9]. Subsequently, the droplets are sorted according to fluorescence or color of the product. The coupling of microfluidics with FACS results in the ultrahigh-throughput screening of metagenomic libraries. However this technique has its own limitation that only fluorogenic substrates not capable of crossing the oil phase barrier may be used [9]. Sometimes, agarose is included in the droplets to increase the strength of structure, which forms the microfluidic gel microdroplets (GMD) [59].

The GMD technique was employed to co-encapsulate a fluorogenic substrate together with clones from a metagenomic library to screen for unique genes with lipolytic activity. Using this method, a lipolytic enzyme EstT was identified [59]. Additionally, GMD-FACS was also used as an ultrahigh-throughput screening in the detection of novel antibiotics [60]. Clones of metagenomic library constructed from three strains of *Staphylococcus* were co-encapsulated in GMD with target pathogen *Staphylococcus aureus*. The GMD was treated with fluorescent dye SyTox Orange and the fluorescence from the DNA-bound SyTox Orange indicates the antibiotic activity. The screening resulted in the discovery of a lytic hydrolase specific for *S. aureus*.

In order to overcome the limitations associated with microfluidics coupled to FACS, efforts were made to improve the microfluidic devices. To bypass the expensive FACS operation, an ultrahigh-throughput technique based on the water-in-oil droplets was developed [61]. The biomimetic compartments within the droplets act as a genotype phenotype linkage in analogy to cells. Such a method was used to screen for novel hydrolase genes from a large metagenomic library derived from a wide range of sources, with sulfate monoester and phosphate tri-ester as fluorescence substrates. The resulting hydrolases span three protein super-families, most of which have very low sequence homolog with known proteins.

In another study, expression of alkaline phosphatase was carried out by encapsulating single *E. coli* cells in approximately 800-picoliter droplets. The reaction in the droplets was monitored by connecting a photomultiplier to a microscope to detect the presence of the fluorescent product. The catalytic turnover of the substrate was measured at several locations along the microfluidic channel. The enzyme activities at different time points were used to provide time-resolved kinetic measurements [113].

It is noticeable that almost all ultrahigh-throughput micro-droplet techniques rely on the detection of a fluorescent product. In the cases lacking this type of readout, droplet screening becomes impossible. In order to deal with this unsatisfactory situation, a microfluidic absorbance activated droplet sorter (AADS) was developed [114]. AADS is based on the NAD^+^ dependent deamination of amino acids catalyzed by phenylalanine dehydrogenase (PheDH). The reaction does not produce fluorescence. Instead, it is coupled with a reaction producing an absorbing dye, which triggers the optical sensor next to the microfluidic chip and diverge the flow of droplets. Such method achieved the enrichment of active variants of PheDH up to 2800 folds. However, due to the low sensitivity of absorbance detection, the sorting speed of this method (100 Hz) is significant lower than those reported for the fluorescent-based method (2000 Hz) [61].

## Conclusion and future perspectives

Metagenomes derived from unculturable microorganisms is a great reservoir for the genes with unknown functions. A variety of function-based metagenomic screening methods have been developed to explore these rich genetic resources, resulting in the identification of large number of novel enzymes with unique metabolic activities. However, there are a number of challenges that hamper the discovery of genes with novel functions, such as poor expression of target genes in the host cells and inefficient activity assays for the gene products. The traditional agar plate screening method suffers from the low sensitivity and low throughput. To overcome such problems, high-throughput methods such as FACS-driven screening, and microfluidics-driven screening have been developed. These methods have greatly expanded toolkits for exploring the vast sequence diversity in the metagenomes. In particular, the microfluidics-based screening has shown its potential to screen over 10^7^ variants per day. When coupled with FACS, the microfluidic devices can greatly expand the scope of high-throughput metagenomic screening. The major bottleneck of such technique is the detection method, which is mostly limited to fluorescent signal. In the future, other detection methods, such as mass spectrometry, nuclear magnetic resonance (NMR) and colorimetric assay, may be combined with microfluidic devices to accelerate the discovery of novel biocatalysts or other genes with important functions in the microbiota. On the other hand, most function-based approaches for metagenomic screening are hindered by the biased and insufficient expression in *E. coli*. There is an urgent need to develop a greater range of alternative hosts with good expression of foreign genes of metagenomic origins. To this end, it is essential to develop efficient shuttle vectors that have extended host ranges so that the metagenomic library carried by these vectors can be expressed in various hosts. Alternatively, the translation profiles in *E. coli* may be altered to accommodate the foreign genes by engineering the ribosome proteins or manipulating the factors involved in transcriptional/translational control.

## Competing interests

The authors have declared no competing interests.
